# Drug Delivery Nanosystems for the Localized Treatment of Glioblastoma Multiforme

**DOI:** 10.3390/ma11050779

**Published:** 2018-05-11

**Authors:** L. Nam, C. Coll, L. C. S. Erthal, C. de la Torre, D. Serrano, R. Martínez-Máñez, M. J. Santos-Martínez, E. Ruiz-Hernández

**Affiliations:** 1 School of Pharmacy and Pharmaceutical Sciences, Trinity College Dublin (TCD), Dublin 2, Ireland; lena@tcd.ie (L.N.); collmerc@tcd.ie (C.C.); dossantl@tcd.ie (L.C.S.E.); santosmm@tcd.ie (M.J.S.-M.); 2 Trinity Biomedical Sciences Institute, TCD, Dublin 2, Ireland; 3 Instituto Interuniversitario de Investigación de Reconocimiento Molecular y Desarrollo Tecnológico (IDM), Universitat Politècnica de València, Universitat de València, 46010 València, Spain; cridela2@qim.upv.es (C.d.l.T.); rmaez@qim.upv.es (R.M.-M.); 4 CIBER de Bioingeniería, Biomateriales y Nanomedicina (CIBER-BBN), 28029 Madrid, Spain; 5 Departamento de Farmacia Galenica y Tecnologia Alimentaria, Facultad de Farmacia, Universidad Complutense de Madrid, 28040 Madrid, Spain; dr.serrano@farm.ucm.es; 6 School of Medicine, Trinity College Dublin (TCD), Dublin 2, Ireland

**Keywords:** drug delivery, glioblastoma multiforme, chemotherapy, local treatment, nanoparticles, theranostics, contrast agents, gene delivery, mesoporous silica nanoparticles

## Abstract

Glioblastoma multiforme is one of the most prevalent and malignant forms of central nervous system tumors. The treatment of glioblastoma remains a great challenge due to its location in the intracranial space and the presence of the blood–brain tumor barrier. There is an urgent need to develop novel therapy approaches for this tumor, to improve the clinical outcomes, and to reduce the rate of recurrence and adverse effects associated with present options. The formulation of therapeutic agents in nanostructures is one of the most promising approaches to treat glioblastoma due to the increased availability at the target site, and the possibility to co-deliver a range of drugs and diagnostic agents. Moreover, the local administration of nanostructures presents significant additional advantages, since it overcomes blood–brain barrier penetration issues to reach higher concentrations of therapeutic agents in the tumor area with minimal side effects. In this paper, we aim to review the attempts to develop nanostructures as local drug delivery systems able to deliver multiple agents for both therapeutic and diagnostic functions for the management of glioblastoma.

## 1. Introduction

Glioblastoma or glioblastoma multiforme (GBM) is a highly malignant form of glioma, which is the tumor associated with neoplastic glial cells in the brain, including oligodendrocytes, astrocytes, and ependymal cells [1]. According to the World Health Organization (WHO), GBM is classified as a grade IV brain tumor, which is the most aggressive variation of the malignancies of the central nervous system (CNS) [2]. GBM is also one of the most prevalent malignant brain tumors, with an incidence rate of about 3.19 per 100,000 people per annum [3]. The etiology of GBM remains unknown, although one of the identified risk factors is the abnormal exposure to ionizing radiation [4]. This disease has a complex genetic expression, including gains of chromosomes 7 and 19, losses of chromosomes 10 and 13, amplification of epidermal growth factor receptor (EGFR) and MDM2, mutation of PTEN, NF1, PDGFRA1, IDH1/2, and deletion of CDKN2A/B [1]. Moreover, the histological characteristics of GBM are quite as diverse as its genetic expression, including increasing mitotic and cellular activity, significant angiogenesis, and necrosis. The shape and size of tumor cells are also highly variable, thus the term *multiforme* [4]. GBM invades within the CNS and rarely metastasizes to distant regions [5]. The common symptoms associated with GBM are headaches, cognitive impairment and personality changes, gait imbalances, incontinence, sensory loss, visual disturbances, seizures, confusion, and delirium. Most of the symptoms are nonspecific, therefore, the disease has the risk to be misdiagnosed as other neurological or psychological disorders, such as dementia, epilepsy, or stroke [4].

GBM possesses a number of unique properties that are associated with its generally poor prognosis, including: (1) a large number of malignant cells that are dormant and may develop rapid resistance to anticancer drugs; (2) glioma has a “crab claw-like” invasion pattern, creating unclear borders between malignant and healthy tissue, thus it is extremely difficult to resect completely the tumor tissue during surgery; (3) the surgical procedure may stimulate the growth of malignant cells; (4) the blood–brain tumor barrier prevents most chemotherapies or other anticancer treatments to reach the brain tumor tissue, resulting in a poor cytotoxic activity and the development of drug resistance [6]. For all these reasons, the survival period for most of the patients with GBM is only approximately 1 year, and only 5% of patients survive longer than 5 years [7].

## 2. Standard Diagnosis and Treatment of Glioblastoma Multiforme

The initial diagnostic approach for patients with suspected GBM is magnetic resonance imaging (MRI), which can determine the size, shape, and location of the tumor [8]. Some advanced MRI techniques—such as diffusion-weighted MRI or dynamic susceptibility contrast MRI—may provide some additional information, including the differentiation between GBM and malignant lymphoma [9], or the prediction of *EGFR* gene amplification [10]. In addition, computed tomography might also be employed to determine the presence of the tumor, although its use in clinical practice for the diagnosis of GBM is not so frequent due to its relative lower resolution in comparison to MRI [11]. Positron emission tomography (PET) imaging—which utilizes 18-fludeoxyglucose and is considered as a standard diagnosis approach for many other cancers—offers little value in the diagnosis of GBM due to the significantly higher glucose uptake of the brain when compared to other organs [12]. However, alternative PET imaging using ^11^C-methionine could be useful as a diagnostic test to predict the prognosis of GBM patients [13].

In terms of biomarkers for diagnosis and monitoring of GBM, isocitrate dehydrogenase (IDH) gene mutation and O^6^-methylguanine DNA methyltransferase (MGMT) hypermethylation have been confirmed to have some prognostic and predictive value. IDH mutation was integrated by the WHO in 2016 for the stratification of GBM into primary (IDH-wildtype) and secondary (IDH-mutant), with differences in prognosis [14]. MGMT status was also included in the clinical guidelines for stratification and prediction of efficacy of chemotherapeutics [15]. Other markers that may play a role in GBM diagnosis include *EGFR* gene mutation and amplification, p53 mutations, PTEN mutations, telomerase reverse transcriptase promoter mutation, or alpha thalassemia/mental retardation syndrome x-linked gene mutation.

Despite all of the above, none of the current diagnosis standards provide real-time dynamic information on tumor progression and therapeutic efficacy, which would be extremely important to monitor the rapid progression of malignant tumors such as GBM [8].

With regards to therapies against GBM, the standard for newly diagnosed patients consists of surgical resection, followed by radiotherapy of the surgical cavity and concurrent chemotherapy [16]. Thus, the initial approach to manage a GBM patient includes surgical debulking, which may alleviate symptoms, and establish the diagnosis by biopsy [5]. During the surgical procedure, Gliadel wafers—a registered product containing cytotoxic drug carmustine (bis-chloroethylnitrosourea, BCNU) incorporated into the biodegradable polymer polifeprosan 20—may be placed in the tumor cavity to slowly deliver the drug to the remaining tumor cells over a period of 3 weeks [17]. In a large phase III clinical trial examining the efficacy of Gliadel wafers, patients treated with placebo wafers had a median survival of 11.6 months, while patients treated with Gliadel had a median survival of 13.9 months [18]. A similar survival gain has been reported with the addition of adjuvant chemotherapy to the post-surgery therapy for GBM, which consists of radiotherapy (60 Gy in 30 fractions). The addition of systemic temozolomide (TMZ, 150–200 mg/m^2^/day continuously for 5 days every 4 weeks) [19] has demonstrated only a limited benefit, with an increase in the median survival of the patients up to 2.5 months [20]. The combined use of Gliadel wafers and TMZ has been associated with frequent adverse effects [17,21]. An additional therapeutic approach, initially approved for recurrent GBM and more recently approved for newly diagnosed patients, consists of tumor-treating fields (TTF) produced by several transducer arrays attached to the shaved scalp of patients. These arrays are connected to an electrical device and generate low-intensity, intermediate-frequency alternating electrical fields that provoke antimitotic effects on malignant cells [22]. A phase III clinical trial comparing TTF therapy plus TMZ with TMZ monotherapy as maintenance treatment in newly diagnosed GBM patients following conventional radiochemotherapy demonstrated a superior outcome with the combination of TTF and TMZ in both progression-free survival (7.1 months vs. 4.2 months in the TMZ-only group) and overall survival (19.4 vs. 16.6 months for the standard arm) [23]. Some concerns regarding the high cost/benefit ratio of this approach have been explained in the literature [24].

The vast majority of GBM patients treated with the standard therapy experience recurrence of the disease, and only about 10% of the total number of these patients currently survive for more than 5 years [25]. However, there is no standard consensus for the treatment of relapsed disease [26]. Re-resection, re-radiation, and alternative dosing schemes of systemic TMZ, other chemotherapy agents such as cisplatin or irinotecan, and anti-angiogenic antibodies have been proposed as strategies for the treatment of recurrent disease, although only modest benefits to the patients have been shown by these treatments [5]. The anti-angiogenic drug bevacizumab has very recently received full approval from the U.S. Food and Drug Administration for the treatment of recurrent GBM [27]. The results of a multicenter phase III trial evaluating the addition of bevacizumab to lomustine chemotherapy indicated an increase in the progression-free survival (4.2 months vs. 1.5 months for the chemotherapy alone). Despite this positive outcome, no significant differences were found in overall survival [28]. Therefore, there is an urgent need to develop novel therapy approaches for GBM, improve the clinical outcomes, and reduce the rate of recurrence and adverse effects associated to current options.

## 3. Barriers in the Treatment of Glioblastoma

**Figure 1:**
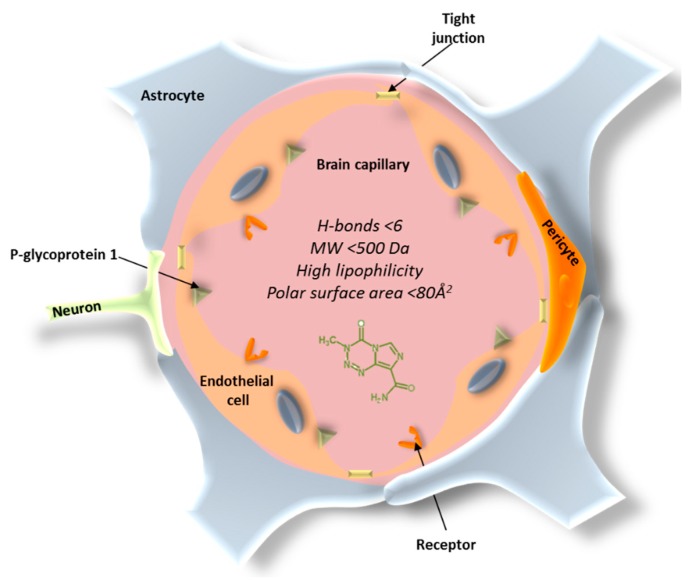
Blood–brain barrier (BBB) structure.

The blood–brain barrier (BBB) constitutes the main obstacle for the systemic treatment of brain tumors and other CNS disorders. The BBB consists of endothelial cells that enclose the brain and spinal cord capillaries and different types of perivascular cells, such as pericytes, astrocytes, microglial cells, and smooth muscle cells (Figure 1) [29]. The main anatomical difference of the BBB endothelial cells is the presence of tight junctions that form a continuous and almost impermeable barrier, resulting in limited paracellular transport of small and lipid-soluble molecules, a lack of fenestrations, and higher mitochondrial content required for the transport of solutes in and out of the brain [30,31]. The complex interactions between these components produce barrier functions that prevent most of the therapeutic compounds to reach the brain. In addition, the substantial presence of P-glycoprotein 1, that can recognize and pump out more than 60% of the marketed drugs, makes the penetration of the compounds into the brain much more difficult [32]. In fact, the BBB only allows the free passage of water, ions, and a small number of lipophilic molecules, while the penetration of large molecules or hydrophilic drugs is often very limited. It has been reported that 98% of small molecules and 100% of large molecules cannot penetrate across the BBB [33].

In addition to the paracellular pathway, there are several routes of transport for chemotherapeutics across the BBB. The transcellular route is associated with low molecular weight and high log D drugs; hence, molecules of less than 500 Da and with high lipophilicity are more favorable to transport across the BBB [34]. However, highly lipophilic drugs can be extensively bound to plasma proteins, resulting in less free available drug, which can compromise brain uptake [35]. The polar surface area is also a key descriptor for BBB permeability. An inverse correlation between polar surface area and brain permeability has been described. When chemotherapeutics possess a polar surface area above 80 Å^2^ and strong capacity to form H-bonds (>6), a higher free energy is necessary to move the molecule from the aqueous environment to the lipid cell membrane of the endothelial cells [34,36,37]. Apart from paracellular and transcellular pathways, other alternative routes for drugs to cross the BBB are receptor-mediated transcytosis (by means of a receptor binding) or adsorptive-mediated transcytosis, which is induced nonspecifically by positively charged molecules [38].

A few disorders and diseases—such as multiple sclerosis, dementia, stroke, autoimmune deficiency syndrome, and brain tumors—may affect the integrity of the BBB. It has been shown that the vascular network of the BBB is disrupted in brain tumors, although the extent of alteration is not likely to result in a massive increase of the amount of drug entering the CNS. Moreover, the invasive nature of high-grade gliomas may produce a widespread presence of malignant cells outside the disrupted region of the BBB [39]. Furthermore, in brain tumor tissues, there is a dense network of tumor vessels—termed as blood–brain tumor barrier (BBTB)—which presents an additional impediment for anticancer drugs to reach malignant tissues. In addition, a large number of efflux transporters expressed on the surface of endothelial cells of tumor tissues may pump the drug out of the cells, thus augmenting the chemotherapy resistance ability of GBM.

Due to all the factors described, there is an insufficient exposure to drugs at the site of action within the brain, and, therefore, the treatment for GBM and other types of intracranial tumors remains a big challenge. Only a small number of cytotoxic drugs, such as TMZ, which possesses an acceptable level of BBB penetration (about 20% of the systemic dose) [40], are currently used to manage high-grade glioma [41]. Despite their promising in vitro cytotoxic activity in GBM cell lines, other drugs, such as doxorubicin (DOX) [42], paclitaxel (PTX) [43], or cisplatin [44], have not been used in standard GBM care due to their poor CNS penetration ability. In these cases, high doses of systemic treatment is required to achieve an optimal concentration at the site of action, resulting in a higher frequency of adverse effects due to the high exposure of healthy tissues to these drugs [45].

## 4. Local Treatment of Glioblastoma

**Figure 2:**
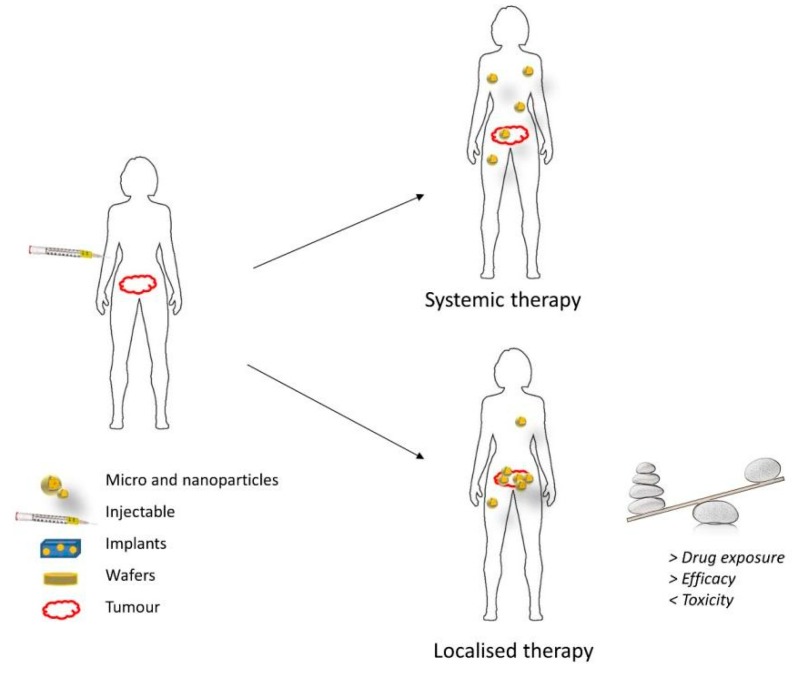
Benefits of localized delivery of chemotherapeutic drugs [46].

Local therapy involves the direct administration of therapeutic drugs that can include chemotherapeutics, immunotherapy, or gene therapy to the tumor location, as opposed to the systemic administration by intravenous injection or oral route. This drug delivery approach has gained significant attention in recent years, as it is thought to circumvent some disadvantages of the systemic administration. Local delivery is expected to increase the amount of drug that reaches the tumor and reduce the systemic exposure of healthy tissues in the body (Figure 2). Thereby, the clinical efficacy of anticancer drugs can be greatly improved, while the incidence of adverse effects is substantially reduced [46]. Localized delivery vehicles can also be designed in the form of depots, which is the pharmaceutical dosage form that can release the active drug over a long period. Localized drug depots present a number of advantages in the delivery of anticancer drugs: they (1) increase the stability of the chemotherapeutic drugs, (2) generate extended and controlled drug release patterns, thus offering better control on drug levels and reducing the number of invasive drug administrations, (3) can incorporate poorly soluble compounds within the depot, (4) decrease the total amount of drug in the formulation, and (5) reduce side effects of chemotherapeutics [47]. In terms of treatment of GBM and other brain disorders, the local delivery approach seems to offer additional benefits, as it is able to bypass the BBB and BBTB, concentrating higher amounts of drug in the malignant tissues [48]. Furthermore, a recent study from Mathios et al. [49] demonstrated that local chemotherapy can potentiate the efficacy of concurrent immunotherapy as well as reinforce the memory response of the host immune system in GBM mice models. This evidence was only obtained for the two standard chemotherapy drugs against GBM, BCNU, and TMZ. However, the result of this study can incentivize more researches and clinical trials focused on local therapy to treat GBM in the near future.

### 4.1. Direct Intratumor Drug Administration

Direct injection of therapies in the tumor resection cavity, in the surrounding brain parenchyma, or in the ventricle appears as the most straightforward approach to locally deliver therapeutic drugs against a brain tumor. Direct injection might be carried out by the use of intraventricular needles with or without repeated infusion via catheter implants connected with a drug reservoir [50]. Various studies and clinical trials have addressed this approach using different anticancer drugs, such as bleomycin [51], BCNU [52], mitoxanthrone [53], and TNF-alpha [54], for the treatment of gliomas. However, the clinical application of this strategy is still very limited for a number of reasons. Firstly, the depth of distribution is only about 3–5 mm from the site of administration due to its dependence on the concentration gradient [50]. In addition, the invasive administration of this approach may generate various associated adverse effects, namely, bleeding, neurotoxicity, and infections, as well as poor clinical efficacy [11].

Convection enhanced delivery (CED) is another approach for local drug delivery that has received increasing attention in recent years. In this technique, a microcatheter connected with a motor-driven pump is inserted within the tumor mass or around the tumor or the resection cavity. The pump generates the pressure to infuse therapeutic drugs at a rate from about 5 to 10 μL/min. Since the drug flow is independent of its passive diffusivity, this method may drive the drug to penetrate longer distances when compared with direct injection [50]. A number of clinical trials applying CED to treat GBM and other associated gliomas have been performed. Lidar et al. [55] initiated a phase I/II trial that used CED techniques in order to intracranially deliver PTX to 15 patients with recurrent GBM and anaplastic astrocytomas. The imaging results showed that 11 out of 15 patients had a positive response to some extent. However, the survival of the patients was only 7.5 months and the adverse effects rate was still relatively high. Another clinical trial reported by Bruce et al. [56] investigated the efficacy of single infusion of topotecan delivered by CED technique in 16 patients with recurrent GBM or grade III gliomas. From the total number of enrolled patients, 69% demonstrated a radiographic response, with 25% having early responses and 44% having late responses. The survival period was 60 weeks, and four patients were still alive at 310 weeks after receiving treatment. Furthermore, CED has been used to deliver large molecules that might not be able to cross BBB, such as oligonucleotides or proteins. The results of the phase II clinical trial reported by Carpentier et al. [57] employed 34 patients with recurrent GBM to assess the efficacy of the CpG oligodeoxynucleotide, a potent immune stimulant. The patient progression-free period was 6.9 months, while the 1- and 2-year survival rates were 24% and 15%, respectively, considerably higher than the rates of comparable studies. In the clinical trial performed by Bogdahn et al. [58], they compared the efficacy of CED therapy of trabedersen—an antisense oligonucleotide that can inhibit the transforming growth factor beta-2—to standard chemotherapy (TMZ or procarbazine/lomustine/vincristine). The endpoint of this study demonstrated that two dose levels of trabedersen (10 and 80 μM) have similar efficacy in comparison with the standard chemotherapy: tumor control rates at 6 months were 14%, 12%, and 15% in the 10 μM, the 80 μM, and standard chemotherapy groups, respectively. In particular, in the group with better prognostic factors (age less than 55 and Karnofsky performance status more than 80%), the 10 μM trabedersen delivered by CED showed a superior 3-year survival rate (40%) when compared to the standard chemotherapy group (13%). The largest published clinical trial to date was the phase III trial investigating CED therapy with cintredekin besudotox (CB), a recombinant chimeric cytotoxin that can kill the glioma cells overexpressing interleukin-13. The study employed 296 randomized patients with recurrent GBM, of which 192 received CB via CED and 104 were implanted with standard Gliadel wafers. However, this study did not demonstrate any statistically significant differences in terms of both clinical efficacy (median survival for CB group and Gliadel group were 9.1 months and 8.8 months, respectively) and safety profiles between the two groups [59].

### 4.2. Wafers and Implants

The formulation of Gliadel is based on the use of polyanhydride poly[bis(*p*-carboxyphenoxy)] propane–sebacic acid (PCPP:SA), which is considered a suitable polymer for the development of local implants because of a number of factors: (1) its hydrophobicity protects drugs from the aqueous environment, (2) the surface erosion pattern allows near zero-order drug release, (3) the polymer is completely biodegradable, (4) the ratio between PCPP and SA can be modified to control the drug release rate, and (5) the polymer can be easily manufactured as drug implant, wafer, sheet, rod, or microspheres [45]. Following the approval of Gliadel, various attempts have tried to design other polymeric implants and interstitial wafers based on the same polymers for the treatment of brain tumors. For instance, Brem et al. [60] prepared PCPP:SA wafers loaded with different amounts of TMZ and tested their biodistribution and efficacy in a 9 L gliosarcoma rat model. In their study, the animals that were implanted with TMZ wafers presented an extended survival of 92 days when compared to control rats (13 days) and rats treated with oral TMZ (22.5 days). In addition, the combination of TMZ wafers with radiation therapy successfully extended the survival of the rat model up to more than 120 days. The same research group incorporated both BCNU and TMZ into the wafers and tested their product in both a 9 L rat glioma model and a chemoresistant F98 glioma mouse model [61]. The results of the efficacy study in F98 indicated that the survival of the group that received BCNU wafer or TMZ wafer, both in combination with radiotherapy, was 120 days, much higher than control groups. Remarkably, 75% of the mice that received the BCNU + TMZ wafer plus radiotherapy were long-term survivors. PCPP:SA implants have also been investigated with drugs that are not routinely used in the treatment of GBM, such as DOX [42] or camptothecin (CPT) [62]. In both studies, the survival of the treatment group was relatively high and significantly longer than the one in the untreated group, with a survival of 69 days in the CPT study and 45 days in the DOX study.

Various local implants able to control the release pattern of chemotherapeutic drugs have been manufactured. Scott et al. [63] developed a microcapsule of poly(l-lactic) acid or liquid crystal polymer (LCP) to locally deliver TMZ to tumors. The number of orifices in the device was designed to control drug release rates. The results of the in vivo efficacy study in a 9 L rat model demonstrated that all the variations in the microcapsules generated additional survival benefits as compared to oral TMZ or control groups, with the highest survival (62 days) in the groups implanted with LCP microcapsules displaying multiple orifices. Furthermore, a microchip system with the ability to deliver multiple drugs or tightly control the drug release patterns was adapted for the local delivery of BCNU to a 9 L glioma rat model [64]. The microchip devices presented excellent antitumor effects, as the median tumor volume of groups treated with the microchip was 87% smaller than in the control group and similar to the group treated with standard BCNU wafer. A more advanced version of the microchip system, known as micro-electromechanical system (MEMS), in which drug release can be activated by an electric pulse, was developed by Masi et al. [65] for intracranial delivery of therapeutic drugs. This research group designed several devices with a different number of membranes and used TMZ as the model drug for the efficacy assays on 9 L rat models. Among the tested groups, the survival was the highest for the treatment group receiving three-membrane activated devices (40 days vs. 13 days for the control group).

## 5. Nanostructures as Alternative Therapeutics for Glioblastoma

Nanostructures are defined as sub-micrometer systems that can act as “individual units” regarding their properties [66]. Due to their unique characteristics, which are not present at the macro or atomic level, nanostructures are considered one of the most promising drug delivery carriers for the treatment of GBM and other CNS disorders [67]. A wide range of their properties, such as size and surface characteristics, can be easily tuned to load and release drug(s) targeting a specific region and in a desirable controlled manner, minimizing potential side effects. In addition, loaded drugs, including both hydrophobic and hydrophilic molecules, can be protected from early degradation and, consequently, increase their circulation half-life. As reported in previous works [41,42], several nanostructures—including liposomes, micelles, solid lipid nanoparticles, polymeric nanoparticles, inorganic nanoparticles or nanogels, among others—have been developed as drug delivery systems and potential diagnostic agents for GBM over the past decades. Different coatings and surface functionalization of nanostructures can be tailored with a variety of targeting moieties to allow specific uptake of the drug carrier by malignant tissues, thus sparing healthy tissues [3].

Two major approaches have been reported to target and accumulate nanostructures within cancer tissues: (i) passive targeting based on the enhanced permeation and retention (EPR) effect, and (ii) active targeting based on the attachment of specific ligands to the surface of the nanostructure in order to recognize and bind to tumor cells [68,69].

### 5.1. Passive Targeting Strategies for GBM

**Figure 3:**
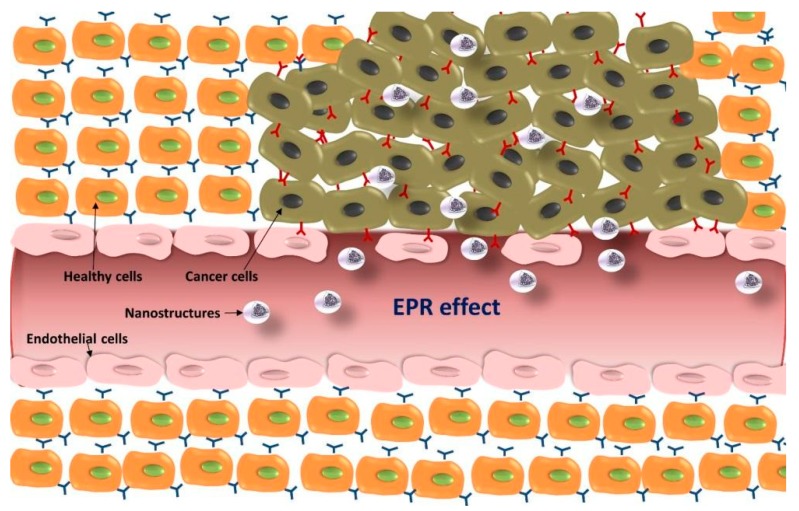
Schematic representation of the enhanced permeation and retention (EPR) effect and accumulation of nanostructures at the tumor site.

One of the advantages of nanostructures for reaching malignant tissues lies in the EPR effect. The vascular network is more vulnerable in tumors than in healthy tissues due to the abnormal development of new blood vessels at the tumor site. Blood vessels from healthy tissues have a pore cutoff size between 4 nm and 25 nm, while the cutoff size in tumor blood vessels can be up to 380–780 nm [70,71]. As a result, systems with sizes up to several hundreds of nanometers can cross those defective blood vessels and reach the surrounding malignant cells (Figure 3). In addition, lymphatic drainage under these circumstances is also impaired and, therefore, those systems can remain at the tumor site for longer periods of time [72]. The size of nanostructures developed for therapeutic purposes can be modified to take advantage of the EPR effect and (1) reach the tumor tissues at a higher rate and (2) reduce their systemic clearance [73].

In relation to the nanostructure size, it is also worth mentioning that nanostructures smaller than 10 nm can be quickly cleared by the kidneys [74], while those between 10 and 100 nm can experience a reduced hepatic filtration and longer circulation periods in the bloodstream [75]. Charge and surface hydrophobicity are also key parameters in order to avoid particle aggregation, opsonization, complement activation, and nonspecific binding to untargeted cells, thereby increasing the efficacy of nanostructures developed for therapeutic applications (specially for highly cationic particles) [76,77,78]. In fact, nanostructures with a zeta potential in the range of ±10 mV and hydrophilic surface (for instance, coated with hydrophilic polymers such as polyethylenglycol (PEG), chitosan, or dextran) tend to have a longer circulation half-life that facilitates accumulation at the tumor site.

Despite the advantages offered by the EPR effect, the passive targeting approach offers a limited benefit in the treatment of gliomas and other CNS disorders. In these situations, the BBB remains impenetrable for a large proportion of nanostructures that tend to accumulate in off-target tissues that also have vasculature gaps such as the liver or lymph nodes. Furthermore, the EPR effect in brain tumors, where the vascular gap ranges from 7 nm to a maximum of 100 nm, is not as relevant as in other tissues [79]. In fact, only a few groups have investigated passively targeted nanosystems for the delivery of drugs to GBM tissues [80]. Steiniger et al. [81] reported the use of a polysorbate-80 coated polybutylcyanoacrylate nanoparticle (PBCA) system loaded with DOX in a 101/8 GBM rat model. The animals treated with loaded nanoparticles exhibited a significant increase in survival and remission time in comparison with the ones treated with empty PBCA, DOX in saline, or DOX in polysorbate-80. The incorporation of DOX into the nanoparticles did not lead to any signs of short-term neurotoxicity. The same research group [82] also tested polysorbate-80 coated poly(isohexyl cyanoacrylate) (PIHCA) nanoparticles loaded with DOX in rats with GBM 101/8 xenografts. In line with their previous observations, histological and immunohistochemical analysis of the animal samples treated with DOX–PIHCA nanoparticles revealed a decrease in tumor size, proliferation index, tumor vessel density, and necrotic areas when compared to the groups treated with empty PIHCA nanoparticles or DOX in solution. Polysorbate-80 coated polybutylcyanoacrylate (PBCA) nanoparticles loaded with TMZ have been also reported to enhance the accumulation of TMZ in brain cancer tissue [83]. In this study, a sustained release of TMZ was observed for PBCA nanoparticles uncoated and coated with polysorbate-80 when compared to free drug in solution. However, although nanoparticles with polysorbate-80 induced a significant increase in the accumulation of TMZ in the brain, high concentrations of drug were also observed in the liver, spleen, and lungs. In another study, curcumin-loaded lipid core nanocapsules (C-LNCs) were designed [84] as a novel strategy to improve the treatment of glioma in vitro, and a sustained release of drug was also observed. When rats bearing C6 orthotopic tumors were treated with C-LNCs, a decrease in tumor size and an increase in animal survival (39 days) was observed when compared to free drug-treated animals (31 days) and saline-treated animals (30 days). In addition, blood compatibility and plasma protein interaction with the C-LNCs systems were also investigated and revealed no signs of hemolysis and protein adsorption, equivalent to the negative control (PBS).

### 5.2. Active Targeting Strategies for GBM

**Figure 4:**
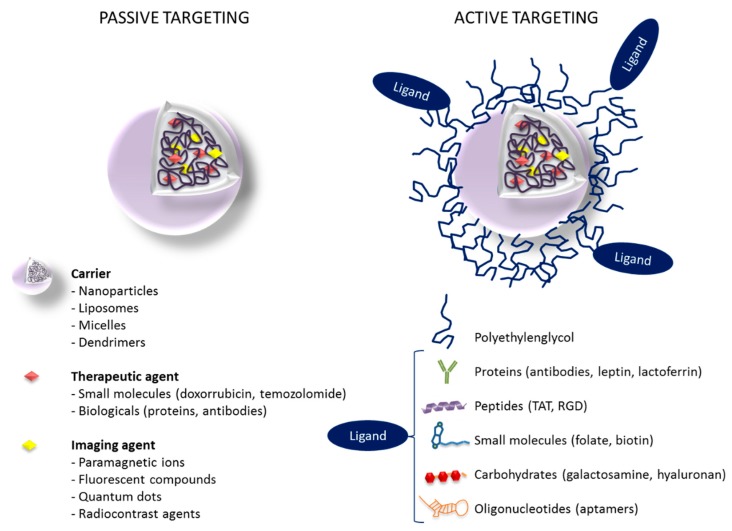
Passive versus active targeting in nanostructures loaded with drugs and imaging agents (theranostics).

Active targeting refers to the use of specific ligands (antibodies, proteins, peptides, nucleic acids) grafted to the surface of the nanostructures to provide a selective binding of the nanostructure to specific receptors or biomolecules that are overexpressed in the tumor and surrounding endothelial tissue (Figure 4). The main targets identified for the treatment of glioma are transferrin (Tf), folate, EGFR, vascular endothelial growth factor (VEGF), αvβ3 integrins, matrix metalloproteinases (MMP), and vascular cell adhesion molecule 1 (VCAM-1) [73]. In the context of GBM and other brain malignancies, active targeting also includes targeting of specific proteins on the BBB capillaries that can potentially play an important role in transferring nanostructures from blood circulation into the brain [85]. In fact, numerous efforts to design actively targeted nanostructures for the treatment of GBM are focused on a dual targeting approach, where the biomolecules grafted to the nanostructure surface may be able to improve both penetration across the BBB and uptake by brain tumor cells [86].

Chlorotoxin (CTX) peptide is one of the most important targeting agents for glioma due to its high affinity for chloride channels and MMP-2 isoforms, which are upregulated in brain cancer tissues. In the study by Fang et al. [87], chitosan (CS) nanoparticles loaded with TMZ and CTX as targeting ligand were synthesized for their application in GBM therapy. It was demonstrated that coincubation of glioma cells (U188, SF767, and GBM6) for 72 h with CTX-conjugated CS nanoparticles loaded with TMZ achieved 2–6-fold higher uptake and 50–90% reduction of IC_50_ values for the drug when compared to coincubation of CS nanoparticles without CTX and free TMZ. In addition, the in vivo study performed in C57BL6 wild-type mice to assess the BBB permeability demonstrated that 2 h after intravenous administration, these CS–TMZ–CTX nanoparticles were widely distributed across the brain tissue, including those parts distal from blood vessels as well as avascular regions.

**Figure 5:**
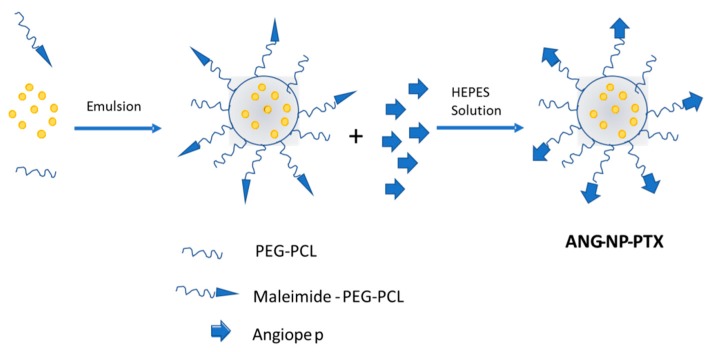
Scheme of the ANG–NP–PTX nanostructure for the dual targeting to brain glioma.

Another important targeting agent to be considered is Angiopep-2 due to its capability to interact with the lipoprotein receptor-related protein (LRP), which is highly expressed in the BBB. In fact, it has been already reported that the use of Angiopep-2 enhances the delivery of genes into the brain through the BBB [88]. LRP is also overexpressed in human glioma cells, and Xin et al. [89] described, for the first time, the use of Angiopep-2 as a dual targeting agent, reporting both an enhanced endocytosis across the BBB and a successful targeting of glioma cells. The final system, ANG–NP–PTX (Figure 5), consisted of Angiopep-2 conjugated to PEG–poly-ε-caprolactone (PEG–PCL) nanoparticles loaded with PTX. The presence of Angiopep-2 conjugated to PEG–PCL showed an increased uptake in U87 MG glioma cells, and while the IC_50_ value for the NP–PTX was 3.4 times lower than for Taxol, it was 3.8 times lower for ANG–NP–PTX than for the NP–PTX. It should be noted that the accumulation of the injected nanoparticles was also detected in the reticuloendothelial system (RES) during the in vivo study.

PTX has been also loaded in PEG–polylactic acid (PEG–PLA) nanoparticles conjugated with EGFP–EGF1 [90]. EGFP–EGF1 is a fusion protein derived from the coagulation factor VII that lacks any procoagulant effect while keeping its binding affinity for tissue factor. It is overexpressed in the neovasculature and most of the tumor cells including glioma cells. The release profile of PTX from nanoparticles with and without EGFP–EGF1 was similar, and no burst release was observed. Uptake studies performed with nanoparticles labeled with coumarin 6 confirmed, by immunofluorescence microscopy and flow cytometry, an efficient uptake and improved cytotoxicity. The efficient penetration of chemotherapy drugs into the tumor site is a key factor to increase the success of the treatment. In a model of GBM cell spheroids, the EGFP–EGF1-conjugated nanoparticles loaded with PTX penetrated 148.9 μm, a greater distance than nanoparticles without fusion protein (93.2 μm). In addition, growth delay was also observed in the glioma spheroids in the presence of the targeted nanoparticles. Further in vivo studies showed that the accumulation of EGFP–EGF1 nanoparticles in glioma tissues was 2.38 times higher than the nontargeted nanoparticles, which mainly accumulated in RES organs. The survival period of mice bearing a C6 glioma xenograft and treated with nanoparticles conjugated with EGFP–EGF1 was significantly longer than the survival of the groups treated with saline, Taxol solution, or nonconjugated nanoparticles (27, 14, 13, and 21 days, respectively).

**Figure 6:**
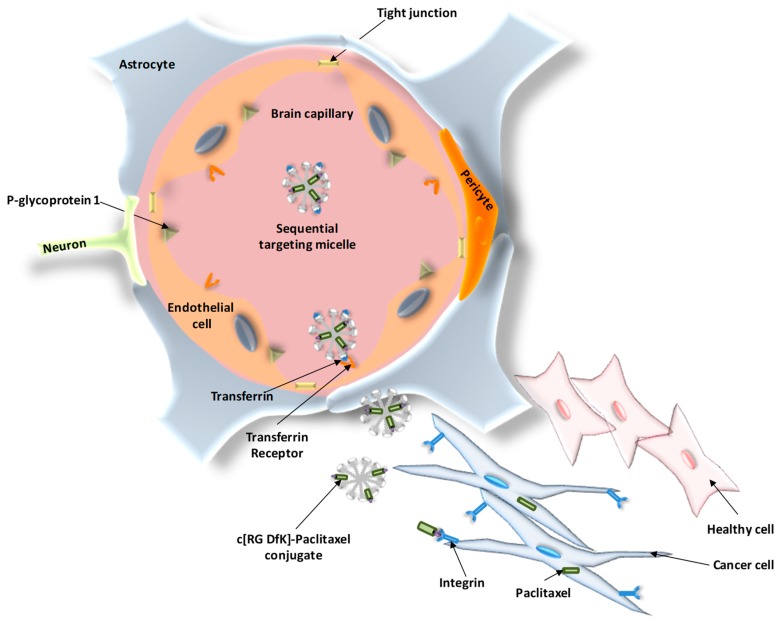
Schematic representation of the sequential BBB penetration and dual glioma targeting of transferrin (Tf)- and c[RGDfK]-modified micelles loaded with paclitaxel.

Tf-conjugated nanostructures have been also widely used for the delivery of cytostatic drugs into the brain by targeting the Tf receptors located at the BBB. An interesting example of dual targeting for sequential BBB penetration and glioma targeting was described by Zhang and coworkers [91]. In their work, a system based on Tf-modified c[RGDfK] PTX conjugate (RP)-loaded micelle (TRPM) was developed (Figure 6). The incorporation of Tf into the external surface of the system increased cell uptake by 2.4-fold, increasing the drug accumulation after intravenous injection and releasing the corresponding RGD conjugate, which was targeted towards the integrin receptor overexpressed in glioma cells. This effect resulted in the accumulation of PTX in glioma and peritumoral tissue. Most importantly, TRPM significantly prolonged the survival time of intracranial glioma-bearing mice (42.8 days) compared to those treated with RPM (39.5 days), PTX-loaded micelle (34.8 days), Taxol (33.6 days), and saline (34.5 days).

In recent years, the interest in gene therapy for cancer treatment has increased and it has become a very promising area of research. In this regard, a safe and efficient gene-delivery method is an essential requirement, and nanostructures are suitable candidates able to overcome biological barriers when administered systemically. Many siRNA delivery systems based on liposomes, silica and polymer nanoparticles, carbon nanotubes, or gold clusters have been reported in the literature [92,93], but there are only a few examples related to brain tumors. A successful gene-delivery system for the treatment of GBM was recently developed by Zarebkohan and coworkers [94] using the plasmid pEGFP, a poly(amidoamine) (PAMAM) dendrimer as carrier, and serine-arginine-leucine (SRL) peptide as the targeting agent. A higher cellular uptake for the PAMAM–SRL material was observed when compared with the PAMAM dendrimer, and when the concentration of PAMAM–SRL material was increased from 0.2 to 1 μM, the uptake raised from 48.8% to 99.7%. The same uptake was obtained with the plasmid-loaded dendrimers. The incorporation of the SRL peptide to the surface of the dendrimer increased the cell viability, reaching the same level as the control or the free peptide. The injection of DNA–PAMAM–SRL polyplexes into mice showed accumulation of the material in the brain, kidneys, and liver. However, no significant accumulation was observed for DNA–PAMAM. A higher *pEGFP* gene expression was observed in the cortical layer for DNA–PAMAM, whereas for DNA–PAMAM–SRL the expression was noted in all parts of the brain without any preference. 

The application of focused ultrasound (FUS) has been proposed as a strategy to improve the transport of nanocarriers through the BBB in a noninvasive and temporary manner [95,96]. Mead et al. used FUS after systemic administration of a colloidal DNA–poly(ethyleneimine) nanoparticle system with a dense PEG coating, named as DNA–BPN [97]. To study the in vivo transfection efficacy using IVIS scanning, DNA–BPN was prepared with a luciferase reporter plasmid. No bioluminescence signal was detected in the CNS outside the FUS local region, and gene expression was not observed in peripheral organs either, including the liver. A dose-dependent transgene expression was observed 24 h post-administration only in the FUS-treated region and lasted for at least 28 days. The transfection efficiency was also determined using DNA–BPN carrying mCherry plasmids driven by the β-actin promoter. One week after FUS treatment, a whole-brain ex vivo epifluorescence imaging confirmed mCherry transgene expression. In the FUS-treated region, around 42% of cells, including neurons and astrocytes, were transfected, while less than 6% of them appeared transfected in the contralateral nontreated hemisphere. No signs of toxicity or astrocyte activation were observed.

All the nanostructures mentioned are suitable systems for the sustained release of drug or oligonucleotides for GBM treatment, given their promising outcomes when compared to the free drug. Although these systems can protect the drug from degradation, reduce its toxicity and be specifically targeted to brain tissue, there is still a risk of premature release as a result of the degradation of the delivery system. For this reason, intensive research is currently focused on the development of stable matrices using nanostructures for the local stimuli-responsive release of drug into the brain, with interesting prospects to improve their applicability.

### 5.3. Drug Delivery Nanosystems Based on Mesoporous Silica Nanoparticles to Treat GBM

**Figure 7:**
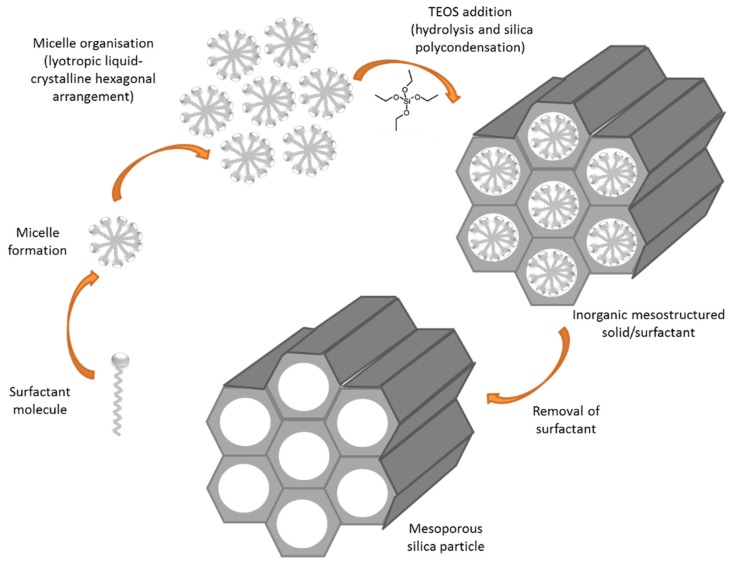
Schematic synthesis of mesoporous silica particles.

Among the highly stable nanomatrices under development, mesoporous silica nanoparticles (MSNs) are being widely explored due to their ability to form the basis of triggered release systems for GBM treatment. MCM41-type MSNs were first described in 1992 as a member of the silicate/aluminosilicate molecular sieves family. These materials are characterized by regular and hexagonal pore arrangements and are synthetized by a procedure called liquid crystal template using surfactants (Figure 7) [98]. By tailoring the surfactant concentration, this synthesis procedure can yield different types of MSNs. Moreover, the surfactant chain length determines the pore size of the material obtained [99].

Due to the easy synthesis and the possibility to adapt some parameters to obtain the desirable product, MSNs have been extensively studied for a broad range of biomedical applications. In terms of nanoparticle size, Mo et al. [100] tested nanoparticles of 20, 40, and 80 nm for their internalization and retention by cells as well as their ability to cross the BBB using a coculture model. The highest level of cell internalization was observed with DOX-loaded MSNs with an average size of 40 nm, which also showed an improved ability to cross the BBB cell model and decreased glioma cell viability. In fact, it was demonstrated that this viability decrease was caused by the overproduction of reactive oxygen species (ROS) and the disruption of the vasculogenic mimicry capacity of glioma cells.

**Figure 8:**
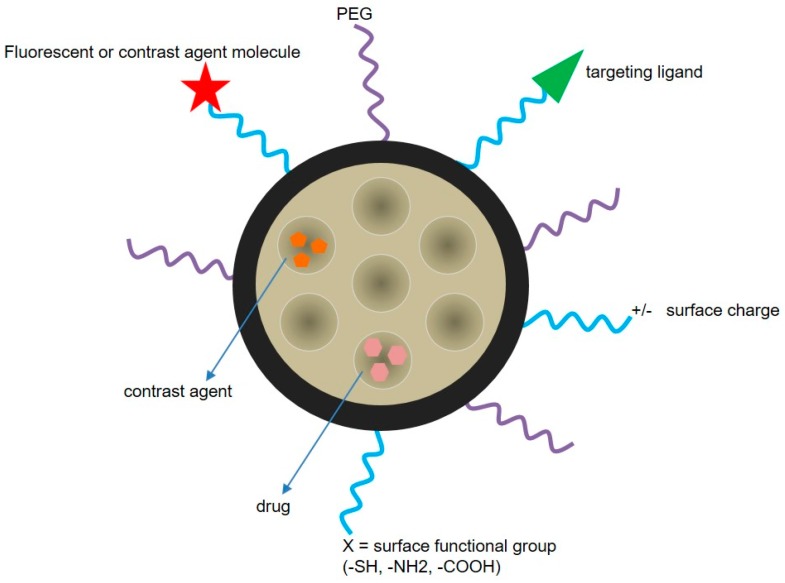
MSN surface can be multi-functionalized to develop a targeting and stimuli-responsive delivery nanosystem. The types of functionalization include the attachment of PEG, fluorescent or contrast agent molecules, and targeting ligands (e.g., protein, peptide, antibody). Surface charge can also be tuned, and the nanoparticles can be loaded with different drugs and/or contrast agents.

**Figure 9:**
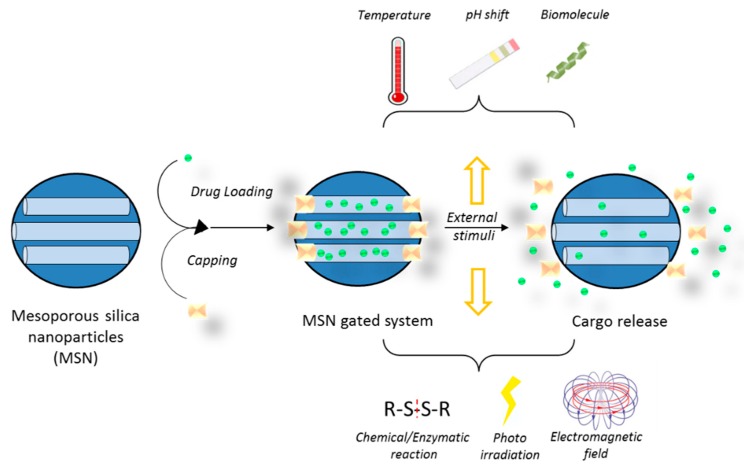
Steps in the development of a gated mesoporous silica nanoparticle system.

MSNs can be simultaneously functionalized with different moieties, which make it possible to combine targets and trigger mechanisms thus increasing the likelihood of a successful therapy (Figure 8). Moreover, the drug cargo can also be bound to the silica matrix in such a way that its release can be triggered by specific tumor cell microenvironment characteristics. Differences in pH between healthy tissues and tumors have been suggested as a mechanism to selectively release chemotherapeutics at the tumor site. Consequently, MSNs able to respond to an acidic pH have been reported for the combined release of CPT and DOX [101]. The nanoparticles were loaded with CPT and surface functionalized with DOX through a hydrazone bond, which is hydrolyzed at pH 6.5. In vitro tests of this system in U87 cells confirmed the synergistic effects between CPT and DOX, as well as the pH-sensitive release. Remarkably, the chemical and structural properties of MSNs make them a very attractive material to be included in diverse triggered release nanodevices [102]. In fact, by using MSNs, it is possible to develop a gated system in which the nanoparticle pores are capped with a certain moiety until the application of a stimulus such as temperature, changes in pH, enzyme activity, or the concentration of reducing agents in the media (Table 1 and Figure 9). Different pore-capping entities have been reported, including polymers, peptides, proteins, antibodies, and inorganic nanoparticles, such as superparamagnetic iron oxide nanoparticles (SPIONs) or quantum dots, among others [103,104,105,106,107]. Ahmadi Nasab et al. [108] used this concept in the design of MSNs loaded with curcumin and coated with a chitosan polymer. The authors demonstrated a lower drug release at pH 7.4 than at pH 5.5 in the presence of the polymer coating. In addition, the IC_50_ of curcumin in U87 cells decreased when delivered by the pH-sensitive nanosystems.

**Figure 10:**
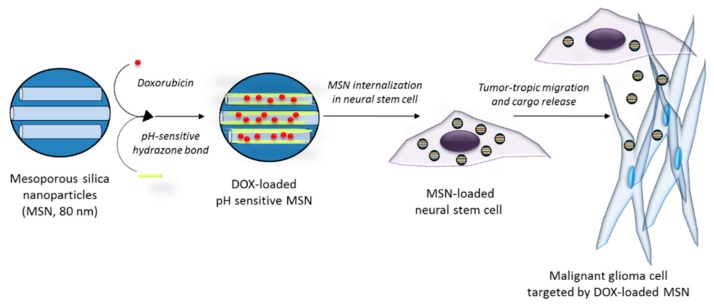
Schematic of malignant glioma cells targeted by neural stem cells carrying a pH-sensitive MSN loaded with DOX.

An interesting approach aiming to improve the targeted drug delivery to tumors by MSNs is the combination of nanoparticles and cells. By integrating the advantages of nanomaterials with stem cell tropism to gliomas, an increase in the efficacy of the therapy is expected. Huang et al. [109] reported the use of a mesenchymal stem cell-based delivery platform in which MSNs were incorporated into stem cells that migrate to the tumor area and promote the localized delivery of the particles. MSNs were coated with hyaluronic acid and tracked through the incorporation of fluorescent dyes and imaging agents, and the accumulation of these MSNs in an orthotopic U87MG GBM xenograft model increased when they were presented by stem cells. In another work, HB1.F3.CD neural stem cells (NSCs) were combined with pH-sensitive MSNs loaded with DOX (Figure 10) [110], and a higher accumulation in a tumor model in vivo was demonstrated in the case of the MSNs carried by NSCs. Moreover, the use of pH-sensitive MSNs prevented DOX toxicity to NSCs and provided a higher efficacy against glioma cells when compared to DOX alone.

In an attempt to enhance the efficacy of radiotherapy against GBM by using MSNs, loading with radiosensitizers has also been proposed. Zhang et al. [111] developed pH-sensitive MSNs loaded with the radiosensitizer valproic acid (VPA) and surface functionalized with folate, which is aimed to target the folic acid receptor overexpressed in cancer cells. VPA-loaded MSNs induced apoptosis in C6 and U87 cell lines after exposure to radiation, and this effect was more pronounced at acidic pH. This design exemplifies the possibility to combine several functions within the same nanoparticle, which makes MSN a promising tool to enhance the diagnosis and treatment of GBM.

### 5.4. Nanotheranostics

“Theranostic” is a term invented by Funkhouser in 2002 to describe an agent that can provide both therapeutic and diagnostic functions [112]. A typical theranostic system usually contains therapeutic drug(s), carrier system, targeting ligands, and a signal emitter that provides the diagnostic function [113] based on different imaging techniques such as fluorescent imaging, ultrasound, MRI, PET, or single photon emission computed tomography (SPECT) [114]. The dual function of theranostics may give rise to the development of precision and personalized medicine in which the patient’s progress and response to therapeutic drugs can be continuously monitored in real time, improving the quality of care and therapeutic outcomes [115].

The unique properties of nanostructures to target and accumulate at tumor tissues constitute a perfect basis for the design of theranostic platforms aimed to treat malignant disorders. Numerous contrast agents for diagnostic imaging display poor water solubility, nonspecific binding, and low stability, thus resulting in short duration of imaging as well as low signal-to-noise ratio. Interestingly, the integration of contrast agents into nanostructures may increase their stability, tissue specificity, and their solubility, which in some cases prolongs imaging duration and increases the resolution of the diagnostic imaging [116]. On one side, nanotheranostics can be used to enhance the therapeutic effect (e.g., radiosensitization) or to allow the application of a specific localized therapy (e.g., photothermal therapy, near-infrared photoimmunotherapy, and magnetic hyperthermia). On the other side, theranostic agents can be understood as probes that enhance tumor imaging, being beneficial for the detection and evaluation of tumor borders, as well as nanoparticle accumulation and signals of therapy outcomes.

In the context of GBM, a variety of nanotheranostic designs has been proposed using different contrast agents and nanocarriers (Table 2). Goel et al. [117] developed MSNs loaded with sunitinib, a tyrosine kinase receptor inhibitor, and surface functionalized with both VEGF_121_ and ^64^Cu. This nanosystem was detected by PET scan images, due to the Cu radionuclide, and showed preferential accumulation in tumor tissue as a result of VEGF receptor targeting. In another approach using MSNs, SPECT was employed to track the nanoparticles that were labeled with ^111^In, and the internalization in NSC enhanced accumulation in a U87 in vivo glioma model [118]. The authors demonstrated that both intracranially and systemically injected cells carrying MSNs reached the tumor site in the brain.

SPIONs have been widely investigated as nanotheranostic carriers due to their small size, ability to provide therapeutic hyperthermia under an alternating magnetic field (AMF), and excellent MRI-enhancing effects. Xu and coworkers [119] evaluated the MRI contrast-enhancing effect and the anticancer efficacy of DOX-loaded SPIONs using a C6 GBM mouse model. In this study, mice treated with drug-carrying SPIONs had longer survival and slower tumor growth rates than the animals treated with saline or free DOX solution. In addition, the application of a magnetic field improved the localization of nanoparticles to glioma cells, and the SPIONs were able to decrease the MRI relaxation time and enhance image contrast. In another study using a T-98 GBM mouse model, Yoo et al. [120] also investigated the MRI-enhancing capability and the drug sensitization effect of SPIONs carrying siRNA of the *MGMT* gene. The authors concluded that the group treated with the combination of TMZ and SPIONs carrying siRNA showed a significant decrease in tumor development rates when compared to the group treated only with TMZ. Additionally, the MRI-enhancing effect of the SPIONs was successfully employed to track the localization of nanoparticles in the glioma cells.

The combination of SPIONs as contrast agents and other nanocarriers has also been explored in previous literature reports. In this way, cell uptake and toxicity of PLGA-based superparamagnetic nanoparticles loaded with TMZ have been studied in C6 glioma cells [121]. The nanoparticles were internalized and the sustained release of TMZ produced a more pronounced decrease in cell viability than the drug alone. Importantly, the nanoparticle presented an MR signal of similar intensity to a commercial contrast agent. Likewise, micelles with both SPIONs and gold nanoparticles, as radiosensitizers, have been prepared and coated with PEG–PCL. The resulting nanotheranostic showed a high degree of sensitization in U251 and U373 GBM cell lines after radiation therapy. When administered intravenously in mice bearing a brain tumor xenograft, it also demonstrated an MRI contrast enhancement up to 120 h and maximum enhancement 48 h after administration [124].

As an alternative MRI contrast agent, gadolinium-based nanoparticles have been suggested to detect, diagnose, and monitor GBM tumors. Gadolinium is already used as contrast agent in the clinic [126], and it has been shown to promote sensitization of tumors to radiotherapy. Gadolinium-based nanoparticles were evaluated by Štefančíková et al., who showed that they enter U87 cells by endocytosis and stay in the cell cytoplasm, colocalizing with lysosomes. As reported by the authors, the effect of these nanoparticles on radiation sensitivity occurs in the cytoplasm and is not related to DNA damage but to ROS generation [127].

The luminescent properties of quantum dots also hold great potential to build nanotheranostic vehicles for the treatment of various cancers. He et al. [125] prepared selenium nanoparticles loaded with CdTe/ZnS quantum dots and ruthenium complexes, and evaluated this nanotheranostic system in different GBM cell lines. This work demonstrated an excellent anticancer effect of the nanosystem on GBM cells via ROS signaling pathways and cell cycle arrest. In order to prove the potential diagnostic performance of the nanoparticles, the authors monitored their intracellular trafficking through the green fluorescent signal emitted by the quantum dots.

As reviewed above, a number of theranostic nanosystems have been explored with significant potential to improve the diagnosis and treatment of GBM. Specifically, the outcomes of localized therapies could be greatly improved if combined with nanotheranostic systems with real-time diagnostic and monitoring capabilities. However, there is still a need to translate the developed materials to the clinical setting. In spite of all the approaches tested and some successful reports in animal models, there is a lack of clinical trials investigating nanotheranostic agents in patients. Therefore, the next steps in this field need to be focused on the safety and efficacy of these nanomaterials in clinical studies.

## 6. Local Treatment with Nanotherapeutics

### 6.1. Intracranially-Administered Drug-Loaded Nanoparticles

Local delivery of nanostructures that carry chemotherapeutic drugs or diagnostic agents is expected to significantly improve treatment efficacy by eliminating issues associated to BBB penetration, increasing the stability of drugs, and reaching higher drug concentrations at the site of action [128]. Çırpanlı et al. [129] used amphiphilic cyclodextrin, PLGA, and PCL nanoparticles to intracranially deliver CPT—a molecule that undergoes rapid hydrolysis when being systemically administered—to 9 L gliosarcoma rat models. Survival analysis showed that the groups treated with cyclodextrin nanoparticles carrying CPT presented an improvement when compared to untreated control groups (up to 33 days vs. 26 days). This result suggests that the intracranial administration of selected nanocarriers may preserve the activity of unstable drugs and improve the clinical outcome. In addition, the surface modification of nanocarriers with PEG may help them bypass the extracellular matrix barrier and penetrate deeper into the tumor tissues [130]. Based on this hypothesis, Nance et al. evaluated PLGA–PEG nanoparticles loaded with PTX (PTX/PLGA–PEG) and compared their efficacy against free PTX and PLGA nanoparticles loaded with PTX (PTX/PLGA) upon CED in a 9 L gliosarcoma rat model. By monitoring tumor size, after 15 days the authors observed a superior performance of PTX/PLGA–PEG, in which the tumor was only 8% of the size in the control group, while the tumor after free PTX and PTX/PLGA was 85% and 45% of the size in the control group, respectively. Zhang et al. [131] followed a similar approach to test cisplatin—a chemotherapy drug that cannot be locally delivered to the brain due to its high toxicity—by CED using poly(aspartic acid) nanoparticles with and without PEG. The results of the efficacy study in a G-98 highly resistant GBM rat model showed 80% survival in the cisplatin-loaded PEGylated nanoparticles group until day 100 post-treatment, while the non-PEGylated nanoparticles group had a survival period of only 40 days. It should be noted that the free cisplatin group had a lower survival period (18 days) than the control group (28 days) due to the high toxicity of the drug. Thus, intracranially administered nanoparticles may not only improve the clinical efficacy, but also decrease the adverse effects of chemotherapeutics.

### 6.2. Gene Delivery

Local administration of nanostructures delivering gene therapy based on miRNA or siRNA seems a promising approach to protect the nucleic acid from degrading factors such as RNases [132], as well as increase the availability of the therapeutic at the tumor site and decrease the adverse side effects associated to systemic administration [133]. Mangraviti et al. [134] formulated poly(β-amino ester) nanoparticles loaded with HSVtk DNA that can act as suicidal gene therapy against GBM. The investigation of CED using these nanocarriers in combination with intraperitoneal ganciclovir in a L-98 glioma rat model showed that the animals receiving the combination therapy had significantly longer survival as compared to intracranial saline, intraperitoneal ganciclovir, or intraperitoneal ganciclovir with CED of naked DNA. Another study by Yu et al. [135] assessed the efficacy of CED and intratumoral injection of 7C1 lipo-polymeric nanoparticles containing a combination of siRNAs that target four major transcription factors promoting the formation of brain tumor-initiating cells in a GBM43 rat model. The authors showed that, although the intratumoral injection and CED of low dose nanoparticles does not offer any survival benefit, the group that received a higher dose by CED prolonged survival for 19 days.

### 6.3. Thermotherapy

Hyperthermia—the heating of cancer tissues between 41 and 45 °C—has shown to improve the efficacy of cancer therapy when used in conjunction with irradiation and/or chemotherapy. Preclinical data demonstrated that the combination of hyperthermia with traditional radio- or chemotherapy regimens improves the efficacy of the treatment without producing additional systemic toxicity, probably due to an enhancement in tumor blood flow. Jordan et al. [136] investigated the efficacy of thermotherapy using aminosilane- and dextran-coated iron oxide nanoparticles in an RG-2 glioma rat model under the action of an AMF. The results showed that dextran-coated nanoparticles were able to raise the temperature to just 39 °C and only slightly improved survival (10.3 days vs. 8.9 days in the control). In contrast, aminosilane-coated iron oxide nanoparticles increased the tumor temperature up to 47 °C and the survival reached 39.7 days. Based on these promising results, a clinical trial of the thermotherapy after administering aminosilane-coated iron oxide nanoparticles intratumorally in humans diagnosed with recurrent GBM was conducted between 2005 and 2009 [137]. Patients underwent standard radiotherapy plus 6 semiweekly thermotherapy sessions with AMF. At the end of the trial, survival from first diagnosis of tumor recurrence was 13.2 months, while survival from primary diagnosis was 23.2 months. Both survival periods were considerably longer than the ones in the standard therapy (6.2 months from first diagnosis of tumor recurrence and 14.6 months from primary diagnosis) [20,25], suggesting the potential of magnetic hyperthermia in combination therapies.

Le Fèvre et al. [122] developed a magnetosome consisting of iron oxide nanoparticles produced by MSR-1 bacteria and coated with poly-l-lysine. In an in vivo study in a GL-261 mice model, the efficacy of magnetosomes versus chemically synthesized iron oxide nanoparticles was compared following the application of an AMF. The results demonstrated that thermotherapy with the use of magnetosomes was superior, with a survival of 147 days, as compared to the iron oxide nanoparticles group (24 days) and the control (15 days). In a similar approach, Ohtake et al. [123] examined μ-oxo *N*,*N*′-bis(salicylidene)ethylenediamine iron [Fe(Salen)] nanoparticles, which possess both intrinsic antitumor activity as well as magnetic properties. The nanoparticles decreased GBM cell viability through ROS generation, and this effect was higher when compared to TMZ and BCNU. In a U251 mouse model, measurements 28 days after intratumoral injection showed that Fe(Salen) nanoparticles managed to decrease tumor size by 50%, and by 90% with the addition of thermotherapy, whereas the tumor size of the control group increased to about 300%. These results reinforce the idea of a combined and localized therapy to achieve better treatment outcomes in GBM.

### 6.4. Theranostics

There have been several attempts to locally deliver nanocarriers containing contrast agents to track their distribution inside the brain by different imaging techniques. Nanosystems based on MRI contrast agents, such as gadolinium in liposomes [138] or SPIONs in PLGA nanoparticles [139], have been developed. Bernal et al. [140] evaluated PLGA nanoparticles loaded with TMZ and iron oxide with theranostic purposes against GBM. Comparing CED and intratumoral injection of the nanoparticles in a rat GBM model, MRI analysis demonstrated that the distribution area is significantly larger in the case of CED. Moreover, the MRI signal of the nanotheranostic did not significantly decrease over a period of 3 weeks. The efficacy assay in a U87 mice model showed that the animals treated with CED of the nanotheranostic had an improved survival when compared to untreated groups and groups treated with empty PLGA nanoparticles. PET imaging has also shown to be feasible for nanostructures locally delivered to the brain. In the study by Zhou et al. [128], PLGA nanoparticles labeled with *N*-(4-[18F]fluorobenzyl)propanamido-PEG4-biotin were delivered to a GBM mouse model by CED. The results of PET imaging were in accordance with postmortem fluorescent microscopy analysis, suggesting that PET can be used as a noninvasive technique to monitor tumor penetration by the nanoparticles.

## 7. Conclusions and Perspectives

Local delivery of chemotherapeutic drugs is considered a feasible approach to effectively manage GBM and other highly malignant brain tumors, since it can bypass the BBB and increase drug availability to tumor tissues. Although a number of reports in the literature and a few clinical trials have investigated the possibility of local delivery with a range of therapeutics, only a very limited number has been approved for clinical practice. One of the main limitations of local delivery modes—such as local injection, drug wafers, and implants—is the short distance of drug diffusion from the site of administration, severely hindering proper contact between chemotherapeutics and tumor tissues. Although CED has been proposed to overcome this limitation by enhancing diffusion into the tumor, it has failed to demonstrate clear benefits over standard therapy. The PRECISE study, the largest clinical trial investigating CED to date, indicated that more than half of the CED intratumoral catheters were improperly placed, which could be one of the reasons for the disappointing results [141]. The optimization of catheter devices and their placement protocols, together with the use of computer modeling for real-time monitoring of placement and infusion processes, are expected to considerably improve the efficacy of CED approaches to treat GBM.

Nanosystems carrying chemotherapeutic drugs or as mediators of alternative therapies offer several advantages over the standard dosing forms in the treatment of GBM. Firstly, nanosystems can protect labile therapeutics from environmental degradation before reaching the target tissues. In addition to raising the concentration of therapeutic or diagnostic agents at the site of action and limiting their systemic clearance, the local administration of nanoparticles or injectable hydrogels can extend and control the release profiles, which open new opportunities for personalized treatments. Secondly, they are able to combine different drugs and diagnostic agents in the same system, with potential synergistic antitumor effects and the possibility to integrate treatment with disease monitoring. As explained by Chiarelli et al. [142], nanostructures are flexible platforms that can incorporate the required building blocks, from metal cores for imaging contrast enhancement and radiation/magnetic therapy to polymeric shells in order to provide biocompatibility and functional sites for the attachment of homing molecules, chemotherapeutics, nucleic acids, or optically active moieties. Thirdly, nanosystem characteristics, such as size, morphology, and surface functionalization, can be tuned with the aim to increase the extracellular matrix penetration and uptake by the tumor tissues.

Despite the promising results in preclinical research using nanoparticles, a limited number of nanomedicines have been approved for clinical practice. Currently, there is only one nanotherapeutic, Nanotherm SPIONs, approved for use in the clinical treatment of GBM, and very few are undergoing phase I and phase II clinical trials [143]. The complexity of some nanoparticle designs, the high production costs, and a significant failure rate in clinical trials have been proposed as possible factors for the low clinical uptake [144]. Research efforts aimed to develop nanotherapeutics for the diagnosis and treatment of GBM should be directed to bridging the gap between preclinical studies and the clinical phase. As a starting point, ongoing research on elucidating the mechanisms of brain tumor growth should be able to reveal novel potential molecular targets for local therapy that could enhance targeting efficiency. Moreover, there is a need to select and optimize the animal models that reflect the heterogeneity of brain tumors in order to efficiently predict therapeutic outcomes and adverse effects of nanotherapeutics. Additionally, reproducibility and scalability of nanoparticle synthetic methods should be improved, and cost should be minimized, to facilitate the translation of novel developments [3], which promises significant advances in the treatment of GBM.

## Figures and Tables

**Table 1:** Examples of actively targeted and stimuli-responsive nanosystems against glioblastoma multiforme (GBM).

Targeted Receptor/Stimuli	Ligand/Responsive Entity	Carrier (Size)	Drug	Beneficial Outcome	Reference
Chloride channel and MMP 2	CTX peptide	CS nanoparticles (<100 nm)	TMZ	Higher uptake (2–6-fold) and IC_50_ reduction (50–90%) in glioma cell lines (U188, SF767, and GBM6) compared to CS nanoparticles without CTX and free TMZ	[79]
LDL	Angiopep-2	PEG–PCL nanoparticles (<100 nm)	PTX	Improved transport across BBB (2-fold higher than Taxol)	[81]
	Serine-arginine-leucine (SRL) peptide	Poly(amidoamine) (PAMAM) dendrimer	Plasmid pEGFP	Increased uptake and accumulation of DNA–PAMAM–SRL system in the brain compared with nontargeted systems	[86]
Tissue factor	EGFP–EGF1 fusion protein	PEG–PLA nanoparticles (<150 nm)	PTX	Longer survival time of glioma-bearing mice (27 days) compared to saline group, Taxol group and nontargeted particles (14, 13, 21 days, respectively)	[82]
Transferrin receptor (Tfr1, also known as CD17)	Transferrin + modified c[RGDfK]	Micelle (98 nm)	PTX	Longer survival time of mice bearing intracranial U87 MG glioma (39.5 days) compared to PTX-loaded micelle (34.8 days), Taxol (33.6 days), and saline solution (34.5 days)	[83]
Acidic pH	Hydrazone bond	MSN	DOX + CPT	Increased drug release at pH 6.5 when compared to pH 7.4, improving the chemotherapeutic effect	[99]
Acidic pH	Hydrazone bond	MSN (80 nm) incorporated into neural stem cells	DOX	Tumortropic migration of neural stem cells carrying DOX-loaded MSN in an intracranial U87 xenograft mouse model, resulting in the induction of apoptosis and improvements in survival (41–42 days) compared to PBS (34 days)	[102]

Key: LDL, low-density lipoprotein receptor; CS, chitosan; TMZ, temozolomide; MMP, matrix metalloproteinases; CTX, chlorotoxin; PTX, paclitaxel; PEG, polyethylenglycol; PCL, poly-ε-caprolactone; TF, tissue factor; EGFP–EGF1, fusion protein derived from factor VII which retains the special binding affinity for TF without inducing coagulation; MSN, mesoporous silica nanoparticles; DOX, doxorubicin; CPT, camptothecin.

**Table 2:** Examples of nanotheranostic systems developed to treat GBM.

Nanoparticle Type	Cargo	Surface Functionalization	Contrast Agent	Detection Method	Combined Therapy	Reference
MSN	Sunitinib	VEGF_121_ and ^64^Cu	^64^Cu	PET	-	[117]
MSN	-	In^111^	In^111^	SPECT and fluorescence microscopy	-	[118]
SPION	DOX	-	Iron oxide	MRI	Magnetic hyperthermia	[119]
SPION	TMZ and siRNA of the MGMT gene	-	Iron oxide	MRI	-	[120]
PLGA-SPION	TMZ	PLGA coating	Iron oxide	MRI	-	[121]
SPION	-	Lysine coating	Iron oxide	MRI	Magnetic hyperthermia	[122]
Fe(Salen) nanoparticles	-	-	Iron oxide	MRI	Magnetic hyperthermia	[123]
Micelles	SPION and Au nanoparticles	PEG-PCL coating	Iron oxide	MRI	Radiotherapy	[124]
Selenium nanoparticles	CdTe/ZnS quantum dots and ruthenium complexes	-	Quantum dots	Fluorescence	-	[125]

Key: MSN, mesoporous silica nanoparticle; SPION, superparamagnetic iron oxide nanoparticle; PLGA, poly(lactic-*co*-glycolic acid); Fe(Salen), μ-oxo *N*,*N*′-bis(salicylidene)ethylenediamine iron; DOX, doxorubicin; TMZ, temozolomide; PEG-PCL, polyethylenglycol-poly-ε-caprolactone; PET, positron emission tomography; SPECT, single photon emission computed tomography; MRI, magnetic resonance imaging.
